# Evaluation of task sharing as a workforce optimization strategy in pediatric oncology

**DOI:** 10.3389/fonc.2025.1560208

**Published:** 2025-04-28

**Authors:** Saman K. Hashmi, Muhammad Rafie Raza, Muhammad Shamvil Ashraf, Ghulam Qadir, Uzma Imam, Zehra Fadoo, Alia Ahmad, Haleema Saeed, Tariq Ghafoor, Nuzhat Yasmeen, Zulfiqar A. Rana, Muhammad Haroon Hamid, Mohammad Fahim ur Rehman, Ameer Ahmad, Rabia Iqbal, Juverya Syed, Sundus Hashmani, Wasfa Farooq, Carlos Rodriguez-Galindo, Sima Jeha, Asim F. Belgaumi, Daniel C. Moreira

**Affiliations:** ^1^ Department of Global Pediatric Medicine, St. Jude Children’s Research Hospital, Memphis, TN, United States; ^2^ Department of Oncology, St. Jude Children’s Research Hospital, Memphis, TN, United States; ^3^ Department of Pediatric Oncology, Indus Hospital and Health Network, Karachi, Pakistan; ^4^ Pediatric Hematology Oncology Service, District Head Quarter Hospital (DHQ) Badin/Indus Hospital and Health Network, Badin, Pakistan; ^5^ Pediatric Oncology Unit, Sheikh Zayed Hospital/Indus Hospital and Health Network, Quetta, Pakistan; ^6^ Children Cancer Centre of Child Aid Association, National Institute of Child Health, Karachi, Pakistan; ^7^ Department of Oncology/Section of Pediatric Oncology, Aga Khan University Medical College, Karachi, Pakistan; ^8^ Paediatric Haematology Oncology Unit, University of Child Health Sciences, The Children’s Hospital, Lahore, Pakistan; ^9^ Department of Pediatric Oncology, Shaukat Khanum Memorial Cancer Hospital and Research Center, Lahore, Pakistan; ^10^ Department of Pediatric Oncology, Combined Military Hospital, Rawalpindi, Pakistan; ^11^ Department of Pediatric Medicine/Pediatric Oncology Unit, Pakistan Institute of Medical Sciences, Islamabad, Pakistan; ^12^ Department of Pediatric Oncology, Children’s Hospital and Institute of Child Health, Multan, Pakistan; ^13^ Department of Pediatric Medicine, King Edward Medical University, Lahore, Pakistan; ^14^ Department of Hematology Oncology, Children’s Hospital and Institute of Child Health, Faisalabad, Pakistan; ^15^ Department of Pediatrics, Bahawal Victoria Hospital, Bahawalpur, Pakistan; ^16^ Department of Pediatrics, Lahore General Hospital, Lahore, Pakistan; ^17^ Department of Pediatric Medicine, Mother and Child Hospital, Nawabshah, Pakistan; ^18^ Department of Pediatrics, Dr. Ruth K. M. Pfau Civil Hospital, Karachi, Pakistan

**Keywords:** pediatric oncology, workforce, capacity building, training, task sharing, medical officers, pediatricians

## Abstract

Task sharing is a pragmatic response to the growing shortage of pediatric oncologists globally, especially in low- and middle-income countries (LMICs). However, there have been limited evaluations of how task sharing has been implemented. In this study, we sought to determine the roles and responsibilities of task-sharing physicians (TSPs) in one LMIC, Pakistan. A multicenter cross-sectional study was conducted across 16 hospitals with secondary- to quaternary-level pediatric oncology facilities. An online survey was used to determine task-sharing models, the responsibilities of TSPs, and the level of supervision. Pediatric oncologists were present at 13 of the 16 centers, with a median of 2 pediatric oncologists per center. We found that TSPs included tiers of medical officers/general physicians and pediatricians. They provided inpatient, outpatient, overnight and emergency room coverage. TSPs could participate in defining cancer diagnosis and risk-stratification (n = 9; 56%), selecting initial chemotherapy plans for patients with newly diagnosed cancer (n = 6; 38%) and modifying chemotherapy on the basis of toxicities (n = 6; 38%) under supervision of a pediatric oncologist. In addition, TSPs could write intravenous chemotherapy orders (n = 10; 63%) and prescribe oral chemotherapy (n = 10; 63%). Furthermore, they could independently perform procedures, such as lumbar punctures (n = 15; 94%), intrathecal chemotherapy administration (n = 11; 69%), and bone marrow aspirates and biopsies (n = 11; 69%). TSPs are critical in the pediatric oncology workforce with responsibilities across the pediatric cancer care continuum.

## Introduction

The overall survival rate for childhood cancer has exceeded 80% in high-income countries (HICs). However, this drops to around 30% in low- and middle-income countries (LMICs), where nearly 90% of children with cancer live (1, 2) and where health systems are not adequately equipped for the complexities of cancer care (3–5). A global shortage in human resources for health reduces access to quality care for the growing burden of childhood cancer (1, 2, 6). Although graduate medical education programs in pediatric oncology are of extreme value, they continue to be scarce or absent in many LMICs (7). Thus, in a system where the care of children with cancer is reliant on only pediatric oncologists to provide care for children with cancer, deficiencies will exist.

Task sharing is the process whereby care responsibilities are shared between specialists and non-specialists, under the oversight of specialists (8). Task sharing has been employed as a pragmatic response to health workforce shortages in different regions and for diverse diseases (9–16). However, how task sharing is being implemented in pediatric oncology has not been comprehensively assessed.

Pakistan is the 5^th^ most populous country in the world, with an estimated population of 241 million, 86 million (36%) of whom are younger than 14 years of age (17). Due to the lack of a national pediatric cancer registry, the exact incidence of pediatric cancer is not known. However, it is estimated that 8000-12000 new cases occur every year (18), though anecdotally less than 5000 are being diagnosed. There are currently 55 pediatric oncologists in the country; the ratio of new diagnoses to pediatric oncologists is thus much higher than the recommended number of 50 patients per specialist for LMICs (6, 19). Due to the lack of specialists in many centers, the responsibilities of pediatric oncologists are being assumed by physicians who lack dedicated training in pediatric cancer care.

In this study, we sought to determine the task-sharing models being used in pediatric oncology units in centers across Pakistan and the roles and responsibilities assigned to non-oncology trained physicians in these units.

## Materials and methods

### Context

Pakistan is administratively divided into four provinces, a federal capital territory, and two disputed territories. Pediatric cancer care is limited to a few centers across the country that have marked variability in their pediatric oncology infrastructure (20). In this study, we included all sixteen centers in Pakistan that had dedicated pediatric oncology units. We did not include centers that were seeing patients for only specific services such as radiation or surgery, and private hospitals that were sporadically seeing small numbers of children with cancer. Eight of the centers in our study were hospitals in central cities with established pediatric oncology teams, while the rest were smaller or newer set-ups, primarily in peripheral cities (**Supplementary Figure S1**). This study was part of an ongoing international collaboration for capacity building through workforce training between St. Jude Children’s Research Hospital (SJCRH) and Pakistan, where training opportunities will be created based on this and other assessments.

### Study design and participants

A cross-sectional survey with 36 independent items was designed to determine site-specific models of task sharing (**Supplementary File**). Survey items were initially developed by the first authors (SKH and MRR) and a senior author (DCM), and iteratively revised by the research team. The survey was piloted at 2 institutions and modified before broader use for the centers in Pakistan. The survey included areas of cancer care infrastructure, including the type of hospital, volume of patients, and number of beds, the structure of the pediatric oncology physician workforce, including which physicians were charged with a modified scope of practice, the specific tasks and responsibilities of task-sharing physicians (TSPs), and the level of supervision. Questions about task sharing pertained to areas of clinical coverage, involvement in diagnostic evaluation and chemotherapy writing/prescribing, and expected decision-making knowledge and procedural skills. The survey was created by using Qualtrics (Provo, Utah) and electronically distributed to the department heads of pediatric oncology or pediatric units, as applicable. It was completed between June 2022 and March 2023.

All participants provided consent to participate in the study. The study was approved by the institutional review board at St. Jude Children’s Research Hospital (SJCRH), USA.

### Analyses

Survey responses were included if at least 75% of the items were completed. Descriptive statistics were used to analyze all items. Because of the limited sample size, comparative tests were not conducted, as the primary objective was to provide a broad analysis of task sharing in Pakistan.

## Results

### Pediatric oncology workforce infrastructure

The centers in our study included public and private hospitals with secondary- to quaternary-level pediatric oncology facilities (6). The hospitals varied in size, both in terms of the number of overall beds (195 to 1900) and the number of new pediatric cancer cases per year (n<20 to n>300). All had inpatient pediatric oncology care, and all except one had outpatient clinics. Fifteen of the 16 hospitals were able to administer outpatient chemotherapy infusions; at the hospital without outpatient clinics, inpatient beds were used for outpatient/day chemotherapy infusions (**Table 1**). Pediatric oncologists were present at 13 of the 16 centers, with a median of 2 pediatric oncologists per center (interquartile range [IQR] of 1 to 4). Seven centers had pediatric hematology-oncology fellowship programs with 3-5 fellows per program (**Table 2**). Twelve centers had rotating residents helping with coverage of pediatric oncology patients as well (**Supplementary Table S1**).

**Table 1:** Pediatric oncology infrastructure at participating institutions (N = 16).

Characteristic	Number of institutions (%) or median (IQR) as indicated^a^
Type of hospital	
General (adult and pediatric)	9 (56)
Pediatric only	5 (31)
Women and children	1 (6)
Cancer only (adult and pediatric)	1 (6)
Hospital model of care	
Public	10 (63)
Public/private partnership	3 (19)
Private for-profit	1 (6)
Private not-for-profit	1 (6)
Private charity	1 (6)
Number of institutions that are referral centers	10 (63)
Monthly referrals *(N = 63)*	
<30	3 (30)^b^
30 – 60	4 (40)^b^
61 – 90	0
>90	3 (30)^b^
Total hospital bed count	
<300	3 (19)
300 – 600	5 (31)
600 – 900	0
≥1000	6 (38)
Unknown	2 (13)
Number of hospitals providing inpatient pediatric oncology care	16 (100)
Median daily inpatient census across centers (IQR)	25 (7 – 38)^a,c^
Number of hospitals with pediatric oncology clinics	15 (94)
Median daily outpatient census across centers (IQR)	20 (8 – 50)^a,d^
Number of hospitals with outpatient chemotherapy infusion area	14 (88)
Median daily infusion center census across centers (IQR)	25 (5 – 30)^a,e^
Centers with opioid availability	8 (50)
New cancer diagnoses annually	
<20	1 (6)
20 – 50	4 (25)
51 – 100	1 (6)
101 – 300	4 (25)
>300	6 (38)

IQR, interquartile range.

^a^Value denotes number of institutions, unless specified otherwise as median and IQR.

^b^Percentage adjusted for number of referral centers.

^c,d,e^median and IQR adjusted for missing values: ^c^Daily inpatient census entered as unknown by respondents of three institutions; ^d^Daily outpatient census missing for two centers (entered as 30 clinics per week and 8-10 patients per week for one center each); ^e^At one center, the inpatient area was also used for outpatient chemotherapy infusion; response for the daily outpatient chemotherapy infusion census was not provided.

**Table 2:** Pediatric oncology physician workforce at participating institutions (N = 16).

Physicians seeing pediatric oncology patients	Number of institutions (%), number of providers, or median (IQR) as indicated^a^
Centers with pediatric oncologists	13 (81)
Total number of pediatric oncologists	53^b^
Median number of pediatric oncologists per center (IQR)	2 (1 – 5)
Centers with medical officers (*MBBS; general physicians, either as a career or prior to pursuing* sp*ecialization; gain on the job experience*)	16 (100)
Total number of medical officers	104
Median number of medical officers per center (IQR)	4 (2 – 9)
Centers with registrars (*additional experience after completion of pediatric (residency) training prior to becoming faculty*)	12 (75)
Total number of registrars	38
Median number of registrars per center (IQR)	2 (2 – 4)
Centers with general pediatricians (*assistant professor and higher rank*)	8 (50)
Total number of pediatricians	41
Median number of pediatricians	5 (2 – 6)
Centers with a PHO fellowship (n; %)	7 (44)
Total number of PHO fellows in training currently	26
Median number of fellows per program (IQR)	4 (3 – 4)
Number of programs with rotating residents	12 (75)

IQR, Interquartile range; MBBS, Bachelor of Medicine, Bachelor of Surgery (equivalent to medical doctor (MD) degree); PHO, pediatric hematology/oncology.

a Value denotes number of institutions, unless specified otherwise as median and IQR (values for median (IQR) rounded up to the nearest whole number, with zeros excluded).

b At one center, there were 7 pediatric oncologists with a standard 4-year training and 3 pediatric oncologists with a condensed 2-year training.

### Task-sharing practices in pediatric oncology units

**Figure 1:**
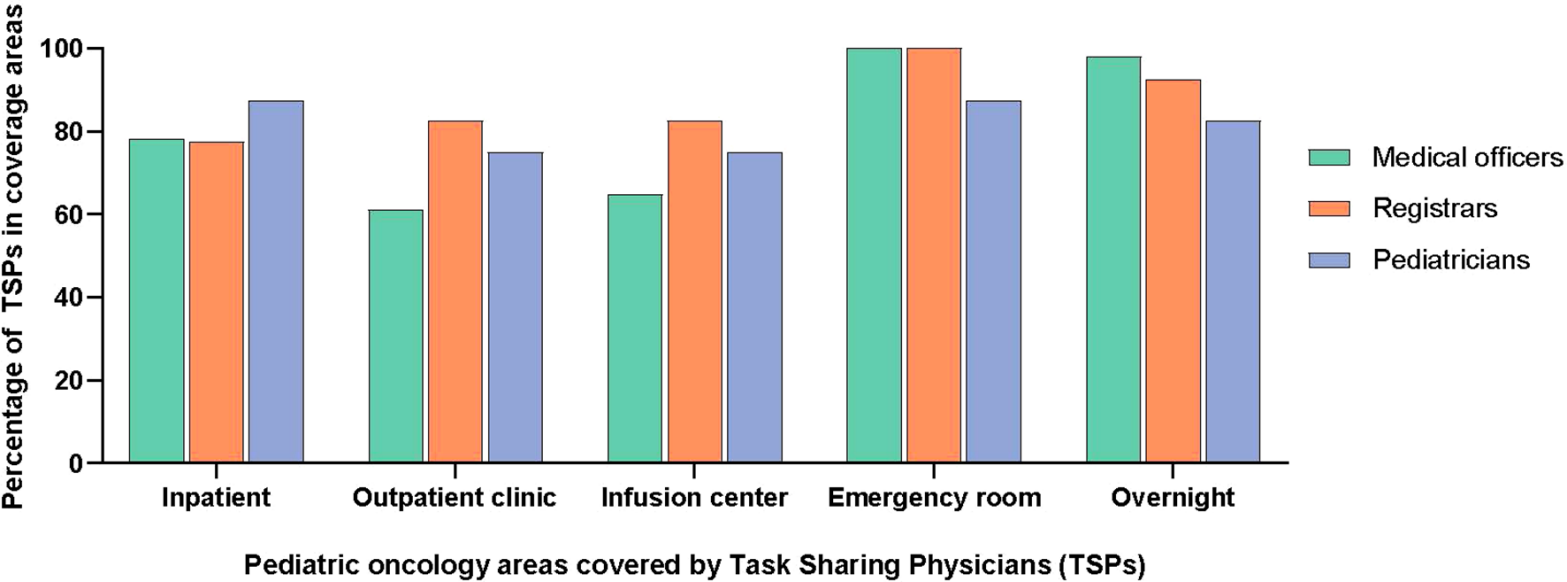
Pediatric oncology coverage by task-sharing physicians.

**Figure 2:**
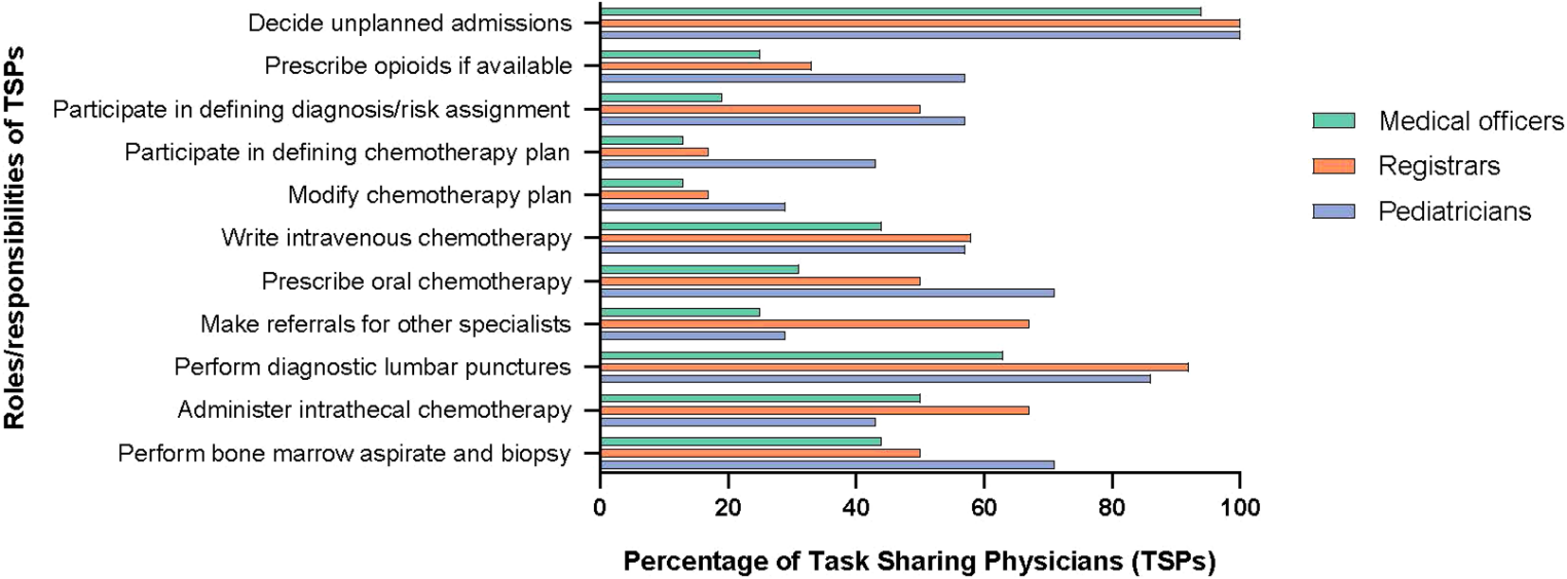
Responsibilities of task-sharing physicians.

Of the non-specialist physicians engaged in pediatric oncology task sharing, medical officers (general physicians) were the most common and were present in all centers, followed by pediatricians. An outline of the physician training model in Pakistan is given in **Supplementary Figure S2**. The TSPs did not have any formal training in pediatric oncology but some centers provided on-the-job “orientation” such as overview of chemotherapy, treatment protocols and supportive care. In terms of clinical coverage, TSPs were managing patients in the inpatient units (n = 13; 81%) and in outpatient clinics/chemotherapy infusion areas (n = 12; 75%). They provided overnight coverage (n = 15; 94%) and triaged patients presenting to the emergency room as well (n = 16; 100%). The details of coverage, roles, and responsibilities of TSPs are included in **Figures 1**, **2**.

In terms of the scope of work, TSPs decided on unplanned admissions, such as those for febrile neutropenia, at all hospitals. At several hospitals, they could participate in defining cancer diagnosis and risk-stratification (n = 9; 56%), selecting initial chemotherapy plans for patients with newly diagnosed cancer (n = 6; 38%) and modifying chemotherapy on the basis of toxicities (n = 6; 38%) under supervision of a pediatric oncologist. In addition, TSPs could write intravenous chemotherapy orders (n = 10; 63%) and prescribe oral chemotherapy (n = 10; 63%). Furthermore, they could independently perform common procedures in pediatric oncology, such as lumbar punctures (n = 15; 94%), intrathecal chemotherapy administration (n = 11; 69%), and bone marrow aspirates and biopsies (n = 11; 69%). At five centers (31%), TSPs were also involved in the preparation and administration of intravenous chemotherapy.

In the inpatient and outpatient setting, patients seen by TSPs were discussed with a pediatric oncologist at most centers (n = 12; 75%), however, direct examination of every patient by a pediatric oncologist occurred at fewer centers (n = 6; 38%). Among peripheral hospitals that were staffed by a TSP without a pediatric oncologist on site, new patients were referred to the hub center. Subsequently, communication about other patients with pediatric oncologists was done through telemedicine to provide support for decision making (**Supplementary Table S2**).

## Discussion

Although anecdotal reports on task sharing exist in pediatric oncology, the different strategies used have not been formally evaluated and reported. In this study, we have comprehensively described the roles and tasks assigned to non-specialists caring for children with cancer in an LMIC, Pakistan. TSPs are engaged in all hospital settings where pediatric cancer patients are cared for and participate in the key elements of the cancer care continuum.

The presented data includes institutions that vary in terms of the type of hospital, pediatric oncology infrastructure, and trained pediatric oncologists, as well as the volume of patients with pediatric cancer. In Pakistan, TSPs contributed to pediatric oncology coverage across all areas of the hospital and were expected to possess a variety of decision-making and procedural skills necessary for the comprehensive care of a child with cancer. This is consistent with the description of tasks distribution in adult oncology and plans to use non-oncologists as primary oncology providers in some settings (11, 15, 21).

Medical officers were the most common TSPs in our study. They are general physicians and have often gained several years of on-the-job experience. Although they have no formal training in oncology, they were considered instrumental by pediatric oncologists in running inpatient and outpatient services, where they were providing direct patient care and doing the bulk of medical record charting, including updating chemotherapy treatment roadmaps. Notably, initial diagnosis, risk-stratification, and defining a treatment plan are arguably the most complex tasks of pediatric oncology in which TSPs were participating. In most instances here, these responsibilities were directly overseen by pediatric oncologists. This dynamic optimizes the time of pediatric oncologists, consistent with the very nature of task sharing (12, 13). Nonetheless, collection of prospective clinical outcomes and patient satisfaction evaluations would be needed to confirm that the quality of provided care is not negatively impacted by TSPs.

In addition to addressing the problem of limited numbers of trained specialists, TSPs become available to institutions in a more cost-effective way due to their salaries being lower than those of consultants. We did not obtain data on cost savings in our survey, however, anecdotally, the salary of a medical officer can be ten times lower than that of a consultant in Pakistan. Cost saving through task shifting or sharing has been reported for other diseases in LMICs (22, 23). Additional studies evaluating the cost effectiveness of task sharing in pediatric cancer units are needed.

To optimize the use of task sharing, professionals must have the necessary competencies to provide quality care for children with cancer. To facilitate this, an effective course based on a structured and systematic design, starting with a targeted needs assessment, is one key element. Our findings contribute to the targeted needs assessment for a course that is being designed for TSPs that will cover the most relevant elements necessary for working with pediatric oncology patients. We are following the 6-step approach described by Kern et al., which is a systematic method that links curriculum development to health care needs and competencies required of the target audience (24). The authors have previously implemented a course using this approach with success (25).

Our study has several limitations. First, our findings reflect task-sharing practices in one LMIC, limiting broader generalizability of our results to other countries. Nonetheless, the varied characteristics of the institutions included in our study could mitigate this limitation. Second, the validity of the assessment tools can be disputed because a preexisting, validated assessment does not exist, and the surveys used were created *de novo*. To mitigate the risk of poor validity and reliability of data, the surveys were created by a pediatric oncologist, reviewed by content experts, and piloted at 2 sites that were not included in the final dataset.

In conclusion, our findings illustrate that TSPs are responsible for many essential steps in the pediatric cancer care continuum, spanning from initial diagnosis and risk stratification to the prescription of chemotherapy. Tiered training initiatives are being planned to support this critical workforce in their roles, which we anticipate will be applicable in other limited-resource settings as well. Future studies will focus on the impact of these initiatives.

## Data Availability

The datasets used and/or analyzed during the current study are available from the corresponding author on reasonable request.

## Supplementary material

Supplementary Figure 1 Map of Pakistan showing participating institutions.

Supplementary Figure 2 Schema of post-graduate medical training in Pakistan.

Supplementary Table 1 The infrastructure of individual institutions and details of their pediatric oncology physician workforce.

Supplementary Table 2 Participants’ comments on oversight of task-sharing physicians.

Supplementary File Study questionnaire.
