# Supplementary material for: Evaluation of task sharing as a workforce optimization strategy in pediatric oncology
Source: Front Oncol. 2025 Apr 28;15:1560208. doi: 10.3389/fonc.2025.1560208 (PMC12066785; doi:10.3389/fonc.2025.1560208)
Supplement: Supplementary Figure 1 — Map of Pakistan showing participating institutions. [file Image1.pdf]

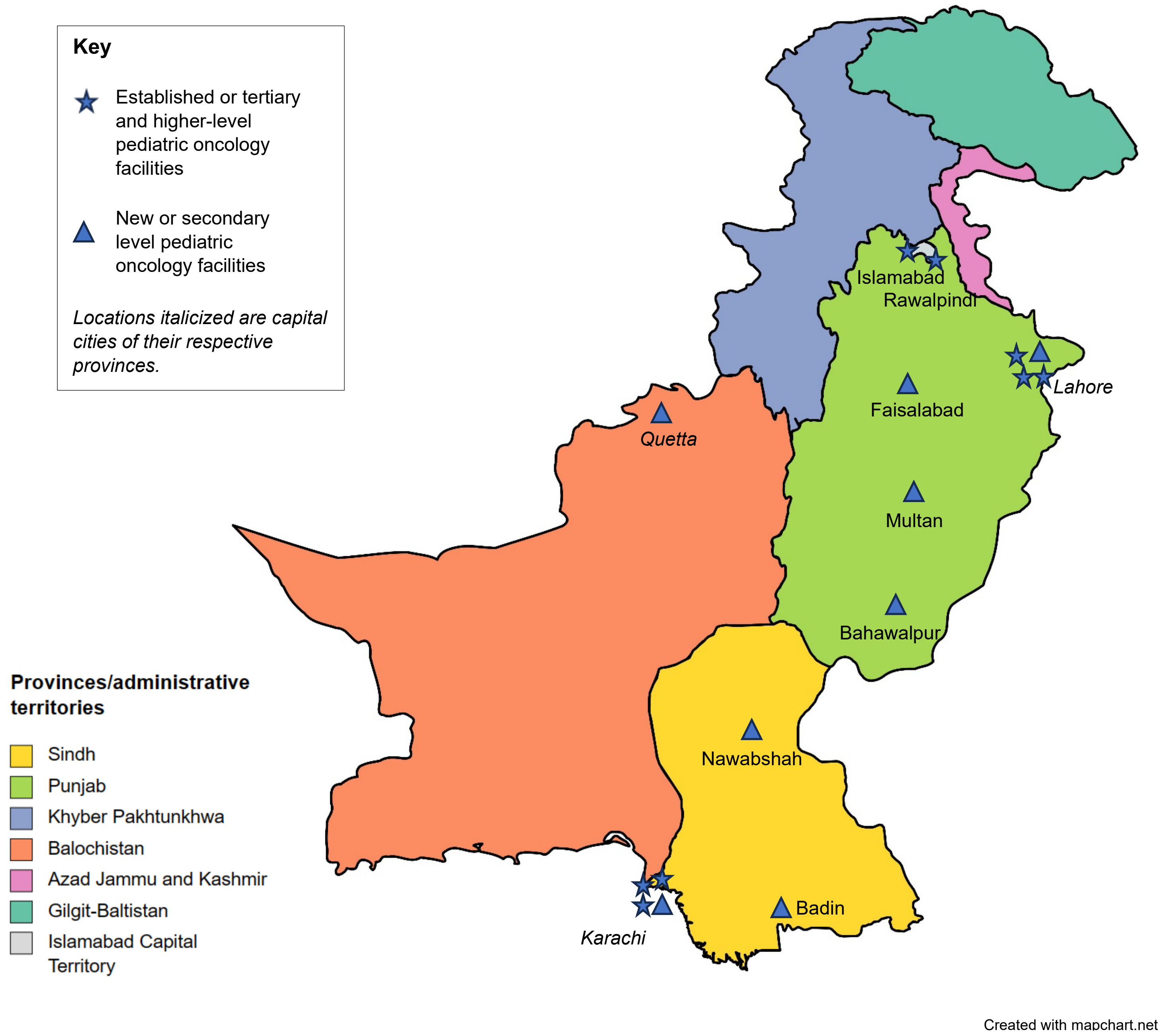

Supplementary Figure S1: Map of Pakistan with locations (cities) of participating institutions
