# Supplementary material for: Evaluation of task sharing as a workforce optimization strategy in pediatric oncology
Source: Front Oncol. 2025 Apr 28;15:1560208. doi: 10.3389/fonc.2025.1560208 (PMC12066785; doi:10.3389/fonc.2025.1560208)
Supplement: Supplementary Figure 2 — Schema of post-graduate medical training in Pakistan. [file Image2.pdf]

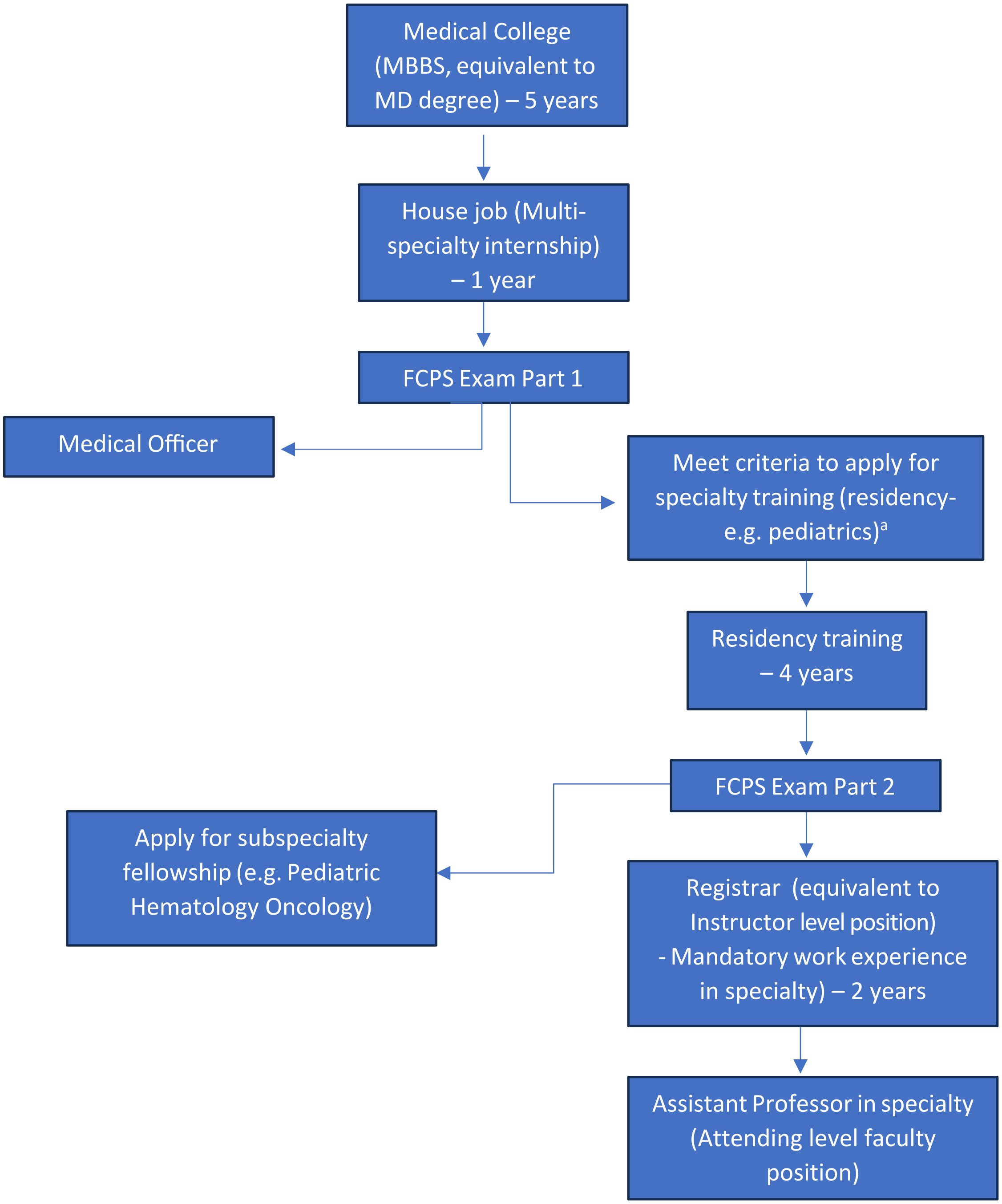

Supplementary Figure S2: Schema of post-graduate medical training in Pakistan

Abbreviations: MBBS (Bachelor of Medicine, Bachelor of Surgery); MD (Doctor of Medicine); FCPS (Fellow of College of Physicians and Surgeons, Pakistan (CPSP)[b]

Footnotes:

[a]The number of subspecialty training positions is much lower than the number of medical graduates. Regulatory authorities have therefore developed scoring criteria (additional examinations/work experience) for eligibility to apply for specialty training.

[b] CPSP is a postgraduate training regulatory body.
