# Supplementary material for: Evaluation of task sharing as a workforce optimization strategy in pediatric oncology
Source: Front Oncol. 2025 Apr 28;15:1560208. doi: 10.3389/fonc.2025.1560208 (PMC12066785; doi:10.3389/fonc.2025.1560208)
Supplement: Supplementary Table 1 — The infrastructure of individual institutions and details of their pediatric oncology physician workforce. [file DataSheet1.docx]

Supplemental Table 1 – Characteristics of individual institutions’ pediatric oncology care

| **ID. No.** | **Public, private or both** | **Total hospital bed count** | **Referral center; monthly referrals** | **Inpatient (ward) daily census** | **Outpatient daily census** | | **New cancer diagnoses annually** | **Pediatric oncologists** | **PHO fellows** | **Rotating residents** | **Medical officers** | | **Registrars** | | **General Pediatric Faculty^a^** |
| --- | --- | --- | --- | --- | --- | --- | --- | --- | --- | --- | --- | --- | --- | --- | --- |
|  |  |  |  |  | Clinic | Chemo infusion area |  |  |  |  | Permanent | Contract | Junior | Senior |  |
| 1 | Public | 200 | Yes; 50 | 10 | 50 | 30 | 101-300 | 1 | 0 | Yes | 0 | 2 | 0 | 1 | 0 |
| 2 | Public/private | 550 | Yes; 40 | 25 | 55 | 30 | >300 | 2 | 0 | Yes | 4 | 0 | 0 | 2 | 2 |
| 3 | Public | 1800 | No | 5 | 3 | 3 | 20-50 | 0 | 0 | Yes | 5 | 3 | 2 | 3 | 7 |
| 4 | Private | 560 | Yes; 15 | 16 | 20 | 55 | 101-300 | 4 | 3 | Yes | 2 | 0 | 0 | 0 | 0 |
| 5 | Public | 1300 | Yes; 100 | 120 | 60 | 130 | >300 | 8 | 3 | Yes | 0 | 2 | 1 | 2 | 0 |
| 6 | Public | 1000 | No | 7 | Area not available | Unknown | 20-50 | 0 | 0 | Yes | 0 | 3^b^ | 0 | 2 | 1 |
| 7 | Public | 300 | No | Unknown (7-bed unit) | 5 | Area not available | 20-50 | 1 | 0 | Yes | 10 | 5 | 3 | 5 | 5 |
| 8 | Public | 1000 | Yes; 25 | 38 | 50 | 10 | >300 | 2 | 4 | Yes | 1 | 0 | 0 | 0 | 0 |
| 9 | Public | 3200 | No | 5 | 8 | 3 | 20-50 | 1 | 0 | Yes | 5 | 2 | 0 | 8 | 6 |
| 10 | Public | 250 | Yes; 30 | 40 | 25 | 15 | 101-300 | 3 | 3 | Yes | 2 | 0 | 0 | 2 | 0 |
| 11 | Private | 195 | Yes; 50 | 34 | 55 | 30 | >300 | 5 | 5 | No | 0 | 20 | 0 | 0 | 1 |
| 12 | Private | 350 | Yes; 120 | 45 | 30 clinics per week | 25 | >300 | 10 | 4 | Yes | 5 | 8 | 0 | 1 | 0 |
| 13 | Public | 300 | Yes; 350 | 30 | 20 | 25 | >300 | 4 | 4 | Yes | 2 | 2 | 1 | 2 | 0 |
| 14 | Public/private | Unknown | No | Unknown | 8-10 patients per week | Area not available | <20 | 0 | 0 | No | 12 | 1 | 1 | 0 | 4 |
| 15 | Public/private | Unknown | Yes; 15 | 8 | 12 | 6 | 101-300 | 1 | 0 | No | 1 | 6 | 0 | 2 | 0 |
| 16 | Public | 1900 | No | 2 | 2 | 2 | 51-100 | 1 | 0 | No | 0 | 1 | 0 | 0 | 15 |

Abbreviation: ID, institution identification number; Chemo, chemotherapy; PHO, Pediatric Hematology Oncology

^a^Assistant Professor level and higher

^b^Comment from respondent: Medical officers rotate off every 3 months; pediatric oncology unit is, thus, primarily overseen by general pediatric consultants, namely a senior registrar and a faculty member.

Supplemental Table 2. Participants’ comments on oversight of task-sharing physicians

| **What type of oversight exists for task-sharing physicians who can write or prescribe chemotherapy?**  - “Chemotherapy prescriptions are rechecked by specialists”  - “The peds oncology faculty/fellow and chemo pharmacy supervises”  - “Dose checking according to weight and CBC report”  - “They work under direct supervision of hem onc consultant”  - “Tumor board” *(response from a center with a pediatrician and 1 pediatric oncology rotating fellow)*  - “Emergency care and steroids and antibiotics can be provided” *(without supervision)*  - “Electronic verification by oncologist”  - “Countersign by consultant”  - “Under observation” *(of a pediatric oncologist)*  - “As they are not fully trained to write oral chemotherapy, *(oncologists*) communicate on telemedicine when they write oral chemotherapy; they need more training”  - “Dose adjustment for oral chemo” *(supervised by pediatric oncologist)*  *- “*They might be unable to reduce dose or modify dose in case of toxicity” |
| --- |
| **Are all patients discussed with a pediatric oncologist? (Yes, No, Other)**  **- “**Before daily ward rounds, chemotherapy planned for daycare patients is discussed with the Oncologist. Those patients who have complaints and those who have derangement in lab parameters are discussed with the Oncologist.”  - “Usually but not always” |
| **Are all patients examined by a pediatric oncologist? (Yes, No, Other)**  - “In-patient examination is performed by Oncologist daily during ward rounds. New patients, patients admitted in PICU, patients coming in clinic without prior appointment but with complaints are also examined. Decision making for difficult situations in Outpatient Clinics is made by Oncologist.”  - “Not all the time, stable daycare patients can come to daycare for chemo, will eventually be seen by oncologist”  - “Some not all patients are seen by consultants”  - “New patients are being referred to Hub center Indus hospital Karachi” *(response from a peripheral district level hospital)*  - “Senior registrars directly evaluate patient and communicate with consultant” |
