# Supplementary material for: Evaluation of task sharing as a workforce optimization strategy in pediatric oncology
Source: Front Oncol. 2025 Apr 28;15:1560208. doi: 10.3389/fonc.2025.1560208 (PMC12066785; doi:10.3389/fonc.2025.1560208)
Supplement: Supplementary Table 2 — Participants’ comments on oversight of task-sharing physicians. [file DataSheet2.docx]

Supplement: Survey

| 1. City: |
| --- |
| 1. Name of institution: |
| 1. Respondent name: |
| 1. Position: |
| 1. Type of hospital: - Selected Choice  - General (adult and pediatric) - Pediatric - Women and Children’s - Pediatric Oncology - Other |
| Type of hospital: - Other (please describe) - Text |
| 1. Is the hospital: - Selected Choice  - Public or governmental - For-profit or private - Not-for-profit - Public/private partnership - Other |
| Is the hospital: - Other (please describe) - Text |
| 1. a. Is your hospital a referral center for pediatric oncology patients?  - Yes - No |
| b. If yes to previous, on average, how many referrals do you receive for pediatric oncology patients from other centers every month? |
| 1. How many total beds does your hospital have? - Selected Choice  - Known (enter bed count) - Unknown |
| 1. Does your hospital have a dedicated pediatric cancer unit? - Selected Choice  - Yes - No - Other |
| Does your hospital have a dedicated pediatric cancer unit? - Other - Text |
| 1. What is the approximate average daily census of inpatient pediatric cancer patients? - Selected Choice  - Known (enter daily census) - Unknown |
| 1. Does your hospital have outpatient services for Pediatric Oncology patients? - Selected Choice  - Yes - No - Other |
| 1. a. Does your hospital have outpatient services for Pediatric Oncology patients? - Other - Text |
| b. If yes to previous, what is your average number of daily outpatient appointments for pediatric oncology patients? |
| 1. Does your hospital have a day chemotherapy infusion/bay? - Selected Choice  - Yes - No - Other |
| 1. a. Does your hospital have a day chemotherapy infusion/bay? - Other - Text |
| b. If yes to previous, what is the average daily census for the infusion room? |
| 1. On average, how many children are diagnosed with cancer at your hospital each year?  - Less than 20 - 20-50 - 51-100 - 101-300 - Greater than 300 - Unsure |
| 1. a. Does your hospital have pediatric oncology specialists (those who have completed dedicated pediatric oncology training)?  - Yes - No - Other |
| b. If yes to pediatric oncology specialist, how many? |
| 1. In terms of physicians *(working in pediatric oncology units)*, how many of the following does your hospital have (enter zero if none)?  - Fellows _____ - Rotating residents _____ - Medical officers (permanent) _____ - Medical officers on contract _____ - Junior registrars _____ - Senior registrars _____ - General pediatricians (completed training in pediatrics; *assistant professor and above*) _____ - Others (specify) _____ |
| If Other is equal to or greater than 1, please specify here |
| *Task sharing is the process whereby care responsibilities are shared between specialists (trained in pediatric oncology) and non-specialists (those who do not have formal training in pediatric oncology) but under the oversight of specialists. The following questions pertain to doctors (‘task sharing professionals’) at your institution who do not have formal training in pediatric oncology (i.e., non-specialists), but are providing care to pediatric oncology patients.* |
| 1. Can task sharing professionals decide admission for a reason that was not planned, such as fever and neutropenia? (Select yes, no, or not applicable)  \|  \| Yes \| No \| Not Applicable \| \| \| --- \| --- \| --- \| --- \| --- \| \| Medical officers \|  \|  \| \|  \| \| Registrars \|  \|  \| \|  \| \| Pediatricians \|  \|  \| \|  \| \| Others \|  \|  \| \|  \| |
| Please specify if yes to others: |
|  |
| 1. Do task sharing professionals cover the inpatient pediatric cancer unit? (Select yes, no, or not applicable)  \|  \| Yes \| No \| Not Applicable \| \| \| --- \| --- \| --- \| --- \| --- \| \| Medical officers \|  \|  \| \|  \| \| Registrars \|  \|  \| \|  \| \| Pediatricians \|  \|  \| \|  \| \| Others \|  \|  \| \|  \| |
| Please specify if yes to others: |
| 1. Do task sharing professionals cover the outpatient pediatric cancer unit? (Select yes, no, or not applicable)  \|  \| Yes \| No \| Not Applicable \| \| \| --- \| --- \| --- \| --- \| --- \| \| Medical officers \|  \|  \| \|  \| \| Registrars \|  \|  \| \|  \| \| Pediatricians \|  \|  \| \|  \| \| Others \|  \|  \| \|  \| |
| Please specify if yes to other: |
| 1. Do task sharing professionals cover the chemotherapy infusion day center? (Select yes, no, or not applicable)  \|  \| Yes \| No \| Not Applicable \| \| \| --- \| --- \| --- \| --- \| --- \| \| Medical officers \|  \|  \| \|  \| \| Registrars \|  \|  \| \|  \| \| Pediatricians \|  \|  \| \|  \| \| Others \|  \|  \| \|  \| |
| Please specify if yes to other: |
| 1. Do task sharing professionals cover the emergency room? (Select yes, no, or not applicable)  \|  \| Yes \| No \| Not Applicable \| \| \| --- \| --- \| --- \| --- \| --- \| \| Medical officers \|  \|  \| \|  \| \| Registrars \|  \|  \| \|  \| \| Pediatricians \|  \|  \| \|  \| \| Others \|  \|  \| \|  \| |
| Please specify if yes to other: |
| 1. Do task sharing physicians cover the pediatric cancer patients on overnight calls? (Select yes, no, or not applicable)  \|  \| Yes \| No \| Not Applicable \| \| \| --- \| --- \| --- \| --- \| --- \| \| Medical officers \|  \|  \| \|  \| \| Registrars \|  \|  \| \|  \| \| Pediatricians \|  \|  \| \|  \| \| Others \|  \|  \| \|  \| |
| Please specify if yes to other: |
| 1. a. Do you have morphine or other opioids available at your institution? |
| b. If yes to previous, can task sharing physicians prescribe opioids? (Select yes, no, or not applicable)   \|  \| Yes \| No \| Not Applicable \| \| \| --- \| --- \| --- \| --- \| --- \| \| Medical officers \|  \|  \| \|  \| \| Registrars \|  \|  \| \|  \| \| Pediatricians \|  \|  \| \|  \| \| Others \|  \|  \| \|  \| |
| Please specify if yes to other: |
| 1. Can task sharing professionals define the diagnosis and risk assignment of children with cancer? (Select yes, no, or not applicable)  \|  \| Yes \| No \| Not Applicable \| \| \| --- \| --- \| --- \| --- \| --- \| \| Medical officers \|  \|  \| \|  \| \| Registrars \|  \|  \| \|  \| \| Pediatricians \|  \|  \| \|  \| \| Others \|  \|  \| \|  \| |
| Please specify if yes to other: |
| 1. Can task sharing professionals define the chemotherapy plan at the start of treatment of children with cancer? (Select yes, no, or not applicable)  \|  \| Yes \| No \| Not Applicable \| \| \| --- \| --- \| --- \| --- \| --- \| \| Medical officers \|  \|  \| \|  \| \| Registrars \|  \|  \| \|  \| \| Pediatricians \|  \|  \| \|  \| \| Others \|  \|  \| \|  \| |
| Please specify if yes to other: |
| 1. Can task sharing professionals modify the chemotherapy plan (dose reductions, for example)? (Select yes, no, or not applicable)  \|  \| Yes \| No \| Not Applicable \| \| \| --- \| --- \| --- \| --- \| --- \| \| Medical officers \|  \|  \| \|  \| \| Registrars \|  \|  \| \|  \| \| Pediatricians \|  \|  \| \|  \| \| Others \|  \|  \| \|  \| |
| Please specify if yes to other: |
| 1. Can task sharing professionals write for intravenous chemotherapy? (Select yes, no, or not applicable)  \|  \| Yes \| No \| Not Applicable \| \| \| --- \| --- \| --- \| --- \| --- \| \| Medical officers \|  \|  \| \|  \| \| Registrars \|  \|  \| \|  \| \| Pediatricians \|  \|  \| \|  \| \| Others \|  \|  \| \|  \| |
| Please specify if yes to other: |
| 1. Can task sharing professionals prescribe oral chemotherapy? (Select yes, no, or not applicable)  \|  \| Yes \| No \| Not Applicable \| \| \| --- \| --- \| --- \| --- \| --- \| \| Medical officers \|  \|  \| \|  \| \| Registrars \|  \|  \| \|  \| \| Pediatricians \|  \|  \| \|  \| \| Others \|  \|  \| \|  \| |
| Please specify if yes to other: |
| 1. What type of oversight exists for task sharing physicians who can write or prescribe chemotherapy? |
| 1. Can task sharing professionals refer patients to other specialists for cancer care (radiation oncology or surgery, for example)? (Select yes, no, or not applicable)  \|  \| Yes \| No \| Not Applicable \| \| \| --- \| --- \| --- \| --- \| --- \| \| Medical officers \|  \|  \| \|  \| \| Registrars \|  \|  \| \|  \| \| Pediatricians \|  \|  \| \|  \| \| Others \|  \|  \| \|  \| |
| Please specify if yes to other: |
| 1. Can task sharing professionals perform diagnostic lumbar punctures? (Select yes, no, or not applicable)  \|  \| Yes \| No \| Not Applicable \| \| \| --- \| --- \| --- \| --- \| --- \| \| Medical officers \|  \|  \| \|  \| \| Registrars \|  \|  \| \|  \| \| Pediatricians \|  \|  \| \|  \| \| Others \|  \|  \| \|  \| |
| Please specify if yes to other: |
| 1. Can task sharing professionals administer intrathecal chemotherapy? (Select yes, no, or not applicable)  \|  \| Yes \| No \| Not Applicable \| \| \| --- \| --- \| --- \| --- \| --- \| \| Medical officers \|  \|  \| \|  \| \| Registrars \|  \|  \| \|  \| \| Pediatricians \|  \|  \| \|  \| \| Others \|  \|  \| \|  \| |
| Please specify if yes to other: |
| 1. Can task sharing professionals perform bone marrow aspirations and biopsies? (Select yes, no, or not applicable)  \|  \| Yes \| No \| Not Applicable \| \| \| --- \| --- \| --- \| --- \| --- \| \| Medical officers \|  \|  \| \|  \| \| Registrars \|  \|  \| \|  \| \| Pediatricians \|  \|  \| \|  \| \| Others \|  \|  \| \|  \| |
| Please specify if yes to other: |
| 1. Are all patients seen by task sharing professionals also directly evaluated by a pediatric oncologist (a pediatric oncologist examines the patient)? - Selected Choice  - Yes - No - Unsure - Other |
| Are all patients seen by task sharing professionals also directly evaluated by a pediatric oncologist (a pediatric oncologist examines the patient)? - Other ( please add comments below) – Text |
| 1. Are all patients seen by task sharing professionals discussed with a pediatric oncologist (a pediatric oncologist discusses the patient, but doesn’t necessarily examine the patient)? - Selected Choice  - Yes - No - Unsure - Other |
| Are all patients seen by task sharing professionals discussed with a pediatric oncologist (a pediatric oncologist discusses the patient, but doesn’t necessarily examine the patient)? - Other (please add comments below) – Text |
|  |
| - How many task-sharing physicians (non-specialists) from your hospital will you be able to provide for a pediatric oncology certification course? |
| - Please provide the names and email addresses of participants from your institution? (Please separate with; between each email address) |

Please click submit to finalize your response. Once submitted, you will not be able to make any changes. You will be able to download a PDF of your responses on the next screen for your reference.

Thank you!
